# The Role of Oxidative Stress and Its Counteractive Utility in Colorectal Cancer (CRC)

**DOI:** 10.3390/cancers12113336

**Published:** 2020-11-11

**Authors:** Debasish Basak, Mohammad Nasir Uddin, Jake Hancock

**Affiliations:** 1 College of Pharmacy, Larkin University, Miami, FL 33169, USA; JHancock@myularkin.org; 2 College of Pharmacy, Mercer University, Atlanta, GA 30341, USA; uddin_mn@mercer.edu

**Keywords:** reactive oxygen/nitrogen species (ROS/RNS), oxidative stress, colorectal cancer (CRC), antioxidant, drug resistance, redox perturbation

## Abstract

**Simple Summary:**

Colorectal cancer (CRC) is very common throughout the world. Despite an improved outcome with the current treatment regimen, a huge number of patients relapse and develop drug resistant disease. Excessive production of reactive oxygen/nitrogen species (ROS/RNS) results in the development of oxidative stress that is closely implicated in the development and progression of CRC. Additionally, the tumor cells can also show an adaptive response against persistent oxidative stress which may lead to chemoresistance. Interestingly, this increased oxidative stress could be manipulated to selectively eradicate the tumor cells. This review depicts how an elevated oxidative stress disrupts different cell signaling pathways and presents the redox modulating agents that showed promising efficacy against CRC derived cell lines.

**Abstract:**

An altered redox status accompanied by an elevated generation of reactive oxygen/nitrogen species (ROS/RNS) has been implicated in a number of diseases including colorectal cancer (CRC). CRC, being one of the most common cancers worldwide, has been reported to be associated with multiple environmental and lifestyle factors (e.g., dietary habits, obesity, and physical inactivity) and harboring heightened oxidative stress that results in genomic instability. Although under normal condition ROS regulate many signal transduction pathways including cell proliferation and survival, overwhelming of the antioxidant capacity due to metabolic abnormalities and oncogenic signaling leads to a redox adaptation response that imparts drug resistance. Nevertheless, excessive reliance on elevated production of ROS makes the tumor cells increasingly vulnerable to further ROS insults, and the abolition of such drug resistance through redox perturbation could be instrumental to preferentially eliminate them. The goal of this review is to demonstrate the evidence that links redox stress to the development of CRC and assimilate the most up-to-date information that would facilitate future investigation on CRC-associated redox biology. Concomitantly, we argue that the exploitation of this distinct biochemical property of CRC cells might offer a fresh avenue to effectively eradicate these cells.

## 1. Introduction

Colorectal cancer (CRC), being the 3rd most common cancer throughout the world, is trending to be a major health problem. In 2018, more than 1.8 million patients were diagnosed worldwide with CRC [1] with approximately 150,000 new cases diagnosed and 52,000 deaths in the United States alone in 2019 [2]. Hence, CRC remains one of the most recurrent and leading causes of death in industrialized western countries. The current treatment approach of CRC comprises of a combination of surgical resection, chemotherapy, radiotherapy, and immunomodulatory therapy. However, nearly 40% of CRC patients eventually relapse and suffer from recurrent or late metastasis making the survival of 5 years feasible for less than 15% of patients [3,4]. An array of unwanted side effects and drug resistance confer the major challenges observed with most of these treatments, thereby abrogating their efficacy. Moreover, the exact mechanisms of CRC development are still poorly understood that indicates an imperative need to comprehend the biology of CRC development better. At the same time, the discovery of novel therapeutic strategies and early diagnostic markers is indispensable [5]. As a result, extensive research is being undertaken to overcome the obstacles of recurrence and drug resistance as well as to explore more effective targets to achieve therapeutic activity and selectivity.

**Figure 1:**
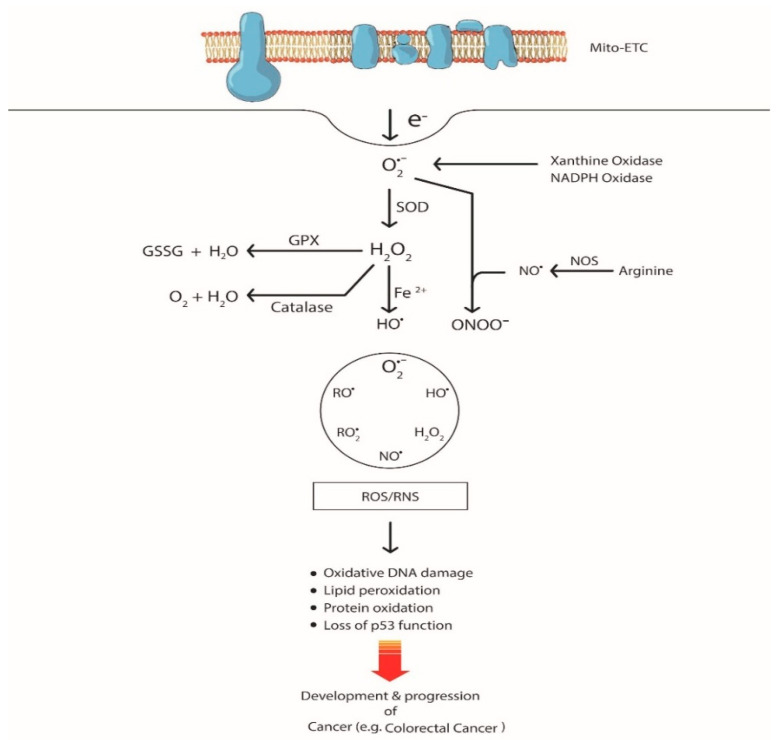
Schematic illustration of reactive oxygen/nitrogen species (ROS/RNS) generation and their link to cancer development. Electron leakage from the mitochondria leads to the generation of superoxide (O•^2−^) by reacting with molecular oxygen. Superoxide, in the presence of superoxide dismutase (SOD), gets converted to hydrogen peroxide (H_2_O_2_). This hydrogen peroxide can be converted either to water by catalase or to hydroxyl radicals (HO•). Arginine, in the presence of nitric oxide synthase (NOS), is converted to nitric oxide (NO•) that reacts with superoxide to form peroxynitrite (ONOO^−^). All these ROS/RNS promote oxidative DNA damage and protein and lipid oxidation. ROS-mediated DNA damage leads to loss of p53 function that results in genomic instability and the development of cancer. GPx, glutathione peroxidase; GSSG, glutathione (oxidized).

Reactive oxygen species (ROS) are oxygen-containing, reactive chemical species that include both the free radicals (e.g., superoxide (O^2•−^), hydroxyl (HO^•^)) and non-radical (hydrogen peroxide (H_2_O_2_)) molecules (Figure 1). Reactive nitrogen species (RNS) originate mainly from arginine with the help of the enzyme nitric oxide synthase (NOS). NOS produces nitric oxide radical NO^•^, which later reacts with O^2•−^ to yield peroxynitrite (ONOO^−^) [6]. They are generated by both enzymatic and non-enzymatic pathways. A non-enzymatic reaction, namely, electron leakage from the mitochondrial respiratory chain, is the major source of ROS. Other enzyme-catalyzed reactions of ROS/RNS production include those involving NADPH oxidase (NOX), xanthine oxidase, β-oxidation in peroxisomes, prostaglandin (PG) synthesis, nitric oxide synthase (NOS), arachidonic acid, and detoxification reactions by cytochrome P450. ROS are essential at a certain level to regulate biological functions and, under normal physiological condition, there exists an equilibrium between oxygen/nitrogen-containing free radicals and antioxidants. This balance gets compromised due to either an elevation in ROS generation or a deficit in antioxidants, which then results in a phenomenon termed “oxidative stress”. Malignant cells harbor endogenous oxidative stress in culture and in vivo compared to the normal counterparts [7,8]. Some of the causative factors of increased oxidative stress are cell culture conditions, sample handling, analytical artefacts, etc. [9,10]. Nevertheless, elevated oxidative stress is highly prominent in different types of cancers as evidenced by the presence of increased levels of oxidized DNA base (8OHdG–8-hydroxy-2′-deoxyguanosine) and lipid peroxidation products in clinical samples [11,12]. In addition to that, altered levels of ROS-scavenging enzymes such as superoxide dismutase (SOD), glutathione peroxidase (GPx), and peroxiredoxin are indicative of aberrant redox homeostasis in tumor cells [13].

**Figure 2:**
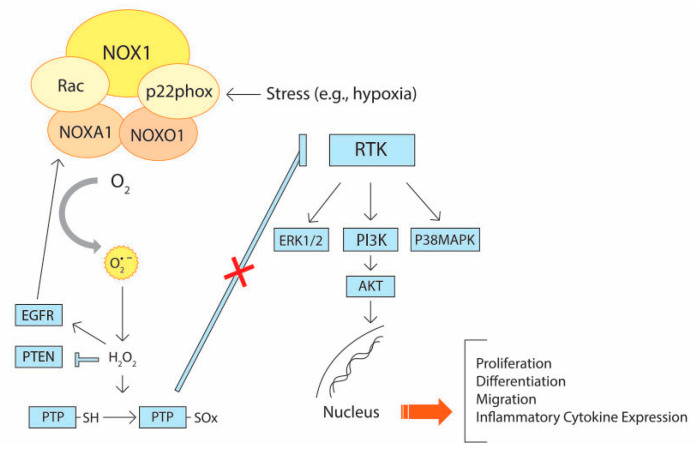
The role of NADPH oxidase (NOX)1 holoenzyme in modulating ROS-mediated intracellular signaling. The activation of NOX1 is elicited by epidermal growth factor receptor (EGFR) and a diverse array of stress factors (e.g., hypoxia). ROS produced by activated NOX1 inactivate protein tyrosine phosphatases (PTPs) by oxidizing the cysteine residues. This results in the stimulation of receptor tyrosine kinases (RTKs) that eventually activate several key signal transduction pathways such as phosphatidylinositol 3-kinase/protein kinase B (PI3K)/AKT signaling and the extracellular signal-regulated kinase mitogen-activated protein (ERK-MAP) kinase cascade. These pathways promote cell proliferation, differentiation, migration, and inflammatory cytokine expression.

An acceleration of glycolysis (the Warburg effect) coupled with an augmented glucose metabolism through pentose phosphate pathway (PPP) in cancer cells results in NADPH generation. Favoring glycolysis over mitochondrial oxidative phosphorylation could decrease the production of ROS (H_2_O_2_ and O^2•−^) that are concomitantly lowered by the redox potential of NADPH [14,15]. Besides intracellular levels, the sources of production and the subcellular accumulation of ROS might trigger opposing cellular responses. Mitochondrial ROS are generally considered to have deleterious activity, whereas the ROS produced by NOX more often triggers some signaling pathways related to cell proliferation. This was clearly illustrated by several studies that used experimental mouse models. For instance, following the loss of adenomatous polyposis coli (APC), ROS produced by the NOX promotes intestinal epithelial cell proliferation, whereas mitochondrial ROS accumulation resulting from TP53-induced glycolysis and apoptosis regulator (TIGAR) depletion decreases cell proliferation [16]. ROS production by NOX1 in the colonic crypts proved to be required for intestinal epithelial cell proliferation [17,18]. ROS production and nuclear factor kappa-light-chain-enhancer of activated B cells (NF-κB) activation triggered by Ras-related C3 botulinum toxin substrate 1 (Rac1) proved to be critical events in CRC initiation [19]. NOX1-induced ROS generation requires the assembly of two cytosolic subunits, namely, NOXO1 (NOX Organizer 1) and NOXA1 (NOX Activator 1) that are homologs of p47phox and p67phox, respectively [20]. NOX1 is highly expressed in colon epithelium and its expression is also dependent on the activation of Rac1 that drives NOX1-mediated ROS generation [21]. A study found that mice lacking NOX1 expression demonstrated impaired angiogenesis and tumor growth, lending support that NOX1 might be crucial in angiogenesis and tumor growth [22]. O’Leary et al. reported that NOX1-mediated redox signaling promotes colon cancer cell adhesion, which consequently facilitates its metastasis [23]. Wang et al. reported NOX1-mediated colon cancer metastasis via activation of the ADAM17 pathway [24]. Juhasz et al. demonstrated that NOX1 facilitates colon cancer cell proliferation by modulating ROS-dependent signal transduction [25]. Very recently, Ohata et al. revealed a major role of NOX1 showing that NOX1-induced ROS stimulates mammalian target of rapamycin complex 1 (mTORC1) activation, which is critical for the proliferation of colon cancer stem cells (CSCs) [26]. Makhezer et al. reported that NOX1-induced ROS upregulate the expression of lipocalin-2 (LCN-2) in inflammatory condition [27]. NOX1-induced ROS contributes to the maintenance of cell proliferation, differentiation, and migration through redox-sensitive cysteine residues [28]. These ROS oxidize the cysteine residues of protein tyrosine phosphatases (PTPs), which leads to the activation of receptor tyrosine kinases (RTKs). This event is associated with the activation of some major signal transduction pathways such as phosphatidylinositol 3-kinase-protein kinase B (PI3K)–AKT signaling and the extracellular signal-regulated kinase (ERK)-mitogen-activated protein (MAP) kinase cascade [29,30]. All these activated intracellular signals upregulate the levels of ROS by further activating NOX1 and thereby regulate cell proliferation, differentiation, migration, and angiogenesis, as summarized in Figure 2.

The development of CRC may be attributed to different factors that include but are not limited to elements of environment and lifestyle (physical inactivity, dietary habit, obesity, etc.). However, a large and compelling body of evidence has suggested a strong correlation between oxidative stress and their link to CRC development and progression as a heightened level of ROS generation was displayed in the chronic diseases of the gastrointestinal tract (GIT) [31]. For example, oxidative stress is a trademark attribute of chronic inflammatory bowel diseases (IBD) such as Crohn’s disease (CD) and ulcerative colitis (UC), which if left untreated facilitate the development of CRC [31,32]. Hence, these GI diseases may participate in CRC in a ROS-dependent way. However, not only oxidative stress but also some other factors can induce tissue damage and facilitate the development of malignancy in chronic inflammation. These may include cytokines, chemokines, eicosanoids, complement components, and different enzymes like collagenase, lipase, phosphatase, and so on [33]. Considering all these, the goal of this review paper is to analyze the relationship between oxidative stress and CRC regarding three points: (1) ROS-mediated genetic alterations, (2) The role of oxidative stress in signaling pathways and transcriptional factor regulation and physiological protective mechanisms against oxidative stress, and (3) Assimilation of redox modulatory agents and their potential to be used in CRC.

## 2. ROS-mediated Genetic Alterations in CRC

### 2.1. DNA Oxidation by ROS

It is now a well-established theory that DNA damages and genomic instability elicited by ROS play central roles in the initiation and progression of different cancers including CRC [34]. ROS-induced primary DNA lesions are single and double-strand DNA breaks. The oxidative nucleobase modifications in DNA that are known to result in carcinogenesis via mispair/mutagenic potential of the modified base include oxidized adenines, thymines, guanines, and cytosines. Other genes that are susceptible to mutations by ROS include p53, V-Raf murine sarcoma viral oncogene homolog B (BRAF), adenomatous polyposis coli (APC), and Kirsten rat sarcoma viral oncogene homolog (KRAS) [35]. One report revealed a direct relation between oxidative stress and p53 mutation in CRC [36]. Compared to normal mucosa, the level of 8-oxodG (8-oxo-7,8-dihydro-2′-deoxyguanosine) is higher in colorectal tumors [37]. This adduct creates G → T transversions during replication and can be metabolized to form 8-oxodGTP, which eventually results in A → C transversions upon incorporation to DNA. The dGTP, being located chiefly in the cytoplasm, is amenable to attack by ROS while dG is shielded by histones due to its presence in the nucleus [38]. Several types of DNA repair enzymes are available that have the potential of repairing the damages induced by 8-oxodG [39]. 8-oxoguanine DNA glycosylase 1 (OGG1) and MutY homolog (MYH) enzymes can be cited as examples that can repair DNA by eliminating the 8-OHdG or mismatched A [40]. However, OGG1 can be negatively regulated by ROS through the oxidation of Cys326 residue [41].

Moreover, mitochondrial DNA is highly vulnerable to ROS damage and is more relevant in CRC [42]. Another striking feature is that DNA damage could generate ROS as well [43]. This was evidenced by the fact that H2A histone family member X (H2AX) displayed NOX1-mediated ROS generation after DNA damage [44]. DNA methylation that plays a central role in gene regulation (overexpression or silencing) has been reported to be compromised when oxidation occurs at either the methylated cytosines or guanines in CpG sequences. The formation of a new adduct obstructs the binding of the DNA methyltransferase to the cytosine residue, resulting in hypomethylation of DNA, a characteristic observed in various cancers including CRC [45]. Concomitantly, the level of antioxidant expression is very robust in CRC that aids their adaptation in a highly oxidized milieu. When this type of redox adaptation gets prolonged, oncogenic signaling becomes activated, leading to carcinogenesis. A study conducted by Van der Logt et al. involved the measurement of ROS production in the whole blood under both unstimulated and phorbol 12-myristate 13-acetate (PMA)-stimulated conditions. Blood sampling was performed at least three months after the surgery of CRC. They concluded that ROS levels under both conditions were significantly higher in patients with a history of sporadic CRC, implicating that ROS may have a pivotal role in the etiology of sporadic CRC [46]. Certain oxidative stress markers have been reported to be found in a greater amount in CRC. These include enhanced levels of overall ROS, 8-oxodG in DNA, lipid peroxides, GPx, and nitric oxide (NO) [47,48,49].

### 2.2. Lipid Oxidation by ROS

ROS-mediated oxidation of polyunsaturated fatty acids (PUFAs) elicits lipid peroxidation, the major products of which are malondialdehyde (MDA) and 4-hydroxy-2-nonenal (HNE) as well as some other minor derivatives such as hydroperoxides, lipoperoxides, conjugated dienes, etc. [50]. Studies showed that oxidative stress is a risk factor of CRC and the levels of MDA and HNE considerably increase in CRC with its staging [51,52]. HNE participates in CRC through dual mechanisms. First, HNE-induced cyclooxygenase-2 (COX-2) activation leads to PG synthesis that stimulates angiogenesis, cell migration and inhibits apoptosis in CRC [53,54]. Second, the upregulation of COX-2 by HNE induces APC loss that activates the wingless-related integration site (Wnt)/β-catenin signaling pathway [55]. Another important mutagen, MDA, stimulates DNA damage by interacting with DNA [56].

### 2.3. Protein Oxidation by ROS

Several intracellular proteins contain thiol (cysteine) residues, and redox modification of these thiols has been reported to regulate numerous protein activities associated with transcription, translation, and biological functions [57]. A good example is the activation of nuclear factor erythroid 2–related factor 2 (Nrf2) through the thiol oxidation of Kelch-like ECH-associated protein 1 (Keap1) [58]. Nrf2 that functions as a major regulator of the cellular antioxidant response is sequestered by Keap1 in the cytosol under normal conditions [59]. Under oxidative stress, thiol oxidation of Keap1 results in the dissociation of Nrf2–Keap1 complex. This ultimately leads to the nuclear translocation of Nrf2, where it regulates the expression of antioxidant genes [60]. The development, progression, and prognosis of CRC involve various redox-sensitive proteins such as phosphatase and tensin homolog (PTEN), transforming growth factor beta (TGF-β), vascular endothelial growth factor (VEGF), and platelet-derived growth factor (PDGF) that necessitate the evaluation of redox status of cysteine residues [61]. The oxidative cysteine modification is mostly carried out by hydrogen peroxide (H_2_O_2_), which is the most prominent intracellular ROS. The stepwise oxidation results in the production of sulfenic acid (R-SOH), sulfinic acid (R-SO_2_H), or sulfonic acid (R-SO_3_H) [62]. All these products except sulfonic acid can be reduced back to thiol state by reducing agents such as thioredoxin, glutaredoxin, peroxiredoxin, and dithiothreitol [63,64]. Hence, the glutathionylation of reactive cysteines is very crucial by which intracellular redox alteration could be transduced into a functional response. However, it is still not clear how thiolation of proteins related to CRC development might be exploited for better therapeutic response and more studies are warranted to discover the exact mechanisms behind this.

## 3. The Impact of Oxidative Stress-Induced Alteration of Signaling Pathways and Transcription Factors in CRC

Several studies have validated the involvement of redox modifications of signaling pathways and transcription factors with the development of CRC. However, in this review, we are going to emphasize three signaling pathways (Janus kinases (JAK)/signal transducer and activator of transcription proteins (STAT), Wnt/β-catenin, and PI3K/AKT pathways) that have been reported to be closely linked to CRC development due to the disruptions by elevated oxidative stress [65,66,67].

### 3.1. Signaling Pathways

The JAK/STAT pathway plays a central role in the growth and survival of CRC cells [65]. A very recent study by Tang et al. showed a positive correlation of colon cancer with the expression of JAK/STAT proteins in terms of clinical stage, tumor infiltration depth, and lymph node metastasis. They recommended the use of JAK/STAT proteins as a diagnostic and prognostic marker for colon cancer [68]. Dimerization of STAT3 through oxidative modification of Cys253 residue activates this signaling pathway by translocating this to the nucleus [69]. However, cysteine modification can also inhibit this pathway as evidenced by STAT3 impairment through S-glutathionylation in Cys328 and Cys542 residues [70]. Apart from thiol modulation, phosphorylation of the Tyr705 residue was reported to activate STAT3 that resulted in the overexpression of cyclinD1 and inhibition of CRC cell apoptosis [71,72]. Finally, redox modification of Cys797 residue in the epidermal growth factor receptor (EGFR) is also reported to activate STAT as well [73].

Another crucial pathway in CRC is Ras-mitogen-activated protein kinase (MAPK) pathway. Oncogenic mutations in BRAF have been reported to upregulate the constitutive activation of MAPK that is attributed to CRC development. It is important to note that KRAS that gets frequently activated by mutations in CRC is upstream of BRAF [74]. The MAPK signaling pathway promotes the phosphorylation and activation of downstream genes associated with CRC [75]. A study revealed the activation of Ras by S-glutathionylation on Cys118 through the elevated levels of ROS [76]. Wnt/β-catenin and Notch signaling pathways also play vital roles in CRC cell proliferation, migration, and differentiation as evidenced by Liu et al. They showed that these pathways are redox-sensitive and can be potentially modulated by NOX [77]. Under normal physiological conditions, these pathways are associated with the maintenance of gastrointestinal homeostasis. In fact, Wnt/β-catenin pathway can be regulated either directly or indirectly through PTEN oxidation by NOX1. Activation of Rac1 drives the assembly of NOXO1 and NOXA1 with NOX1. This NOX1-generated ROS trigger cysteine oxidation of nucleoredoxin (NRX), leading to the dissociation of NRX from Axin-binding protein Dishevelled (Dvl). Dvl mediates the accumulation of β-catenin by inhibiting the degradation of β-catenin complex comprising of Axin, APC, and glycogen synthase kinase-3 (GSK-3) and thereby promotes CRC development [78]. When active, GSK-3 mediates proteasomal degradation of β-catenin through phosphorylation [79]. Conversely, the phosphorylation of GSK-3 by AKT inactivates GSK-3 and targets it for proteasomal degradation [80]. PTEN, being a negative regulator of AKT, can upregulate GSK-3 that can promote the degradation of β-catenin, which ultimately inhibits Wnt signaling. Hence, PTEN can indirectly regulate Wnt signaling [81]. Moreover, PTEN negatively regulates the AKT signaling pathway through the dephosphorylation of phosphatidylinositol (3,4,5)-trisphosphate (PIP3) that is produced by PI3K [82]. HNE, which is a lipid peroxidation product of oxidative stress, was shown to promote angiogenesis in CRC through the overactivation of COX-2 [53]. COX-2 was also reported to activate Wnt/β-catenin signaling pathway by inducing APC loss [55].

PI3K/AKT, another important signaling pathway, was found to be intimately linked to CRC development [83]. A very recent study by Ju et al. reported a novel mechanistic link between redox stabilizing oncogenic signaling and the metabolic adaptation procedures with CRC development. This study found that elevated levels of methylenetetrahydrofolate dehydrogenase (MTHFD2), an NADPH generating enzyme, facilitated CRC cell growth and metastasis when MTHFD2 was transcriptionally upregulated by c-Myc through KRAS downstream effectors, including PI3K/AKT and ERK pathways [84]. Several other studies also lend support to this finding by demonstrating a ROS-mediated activation of PI3K/AKT with subsequent development of CRC [73,83,85,86]. For instance, a study reported ROS-induced migration and invasion of CRC cells through the activation of PI3K-AKT-mTOR signaling pathway [85]. In addition to that, ROS-induced oxidation of Cys124 in PTEN resulted in CRC by activating PI3K signaling [86]. Moreover, EGFR that is amenable to redox modification at Cys797 residue is implicated in PI3K activation [73]. H_2_O_2_ can promote EGFR Tyr phosphorylation through the inhibition of Cys-dependent PTPs [87,88]. An elevated EGFR kinase activity was also evident after the oxidation of Cys797 [89,90]. Essentially, Tyr phosphorylation leads to the activation of MAPK and PI3K/AKT pathways downstream of the EGFR, and these pathways are vital for cell proliferation, invasion, and survival. Thus, the oxidation of PTPs and/or the EGFR results in amplified downstream signaling pathways [91].

The COX pathway that catalyzes the rate-limiting step of PG synthesis from arachidonic acid has also been reported to be linked to CRC development. The overexpression of the inducible isoform COX-2 was reported to be an unfavorable prognostic factor for CRC [92,93]. Concomitantly, its silencing was associated with an attenuation of tumorigenesis and metastatic potential of CRC and other cancers [94,95]. The exact relation between COX-2 activation/suppression and ROS in CRC is not clear. However, some studies have shown an augmented level of oxidative stress upon COX-2 expression. For example, Tesei et al. reported increased oxidative stress along with a heightened COX-2 expression when they had treated human colon cancer cells with nitric oxide-releasing non-steroidal anti-inflammatory drugs (NO-NSAIDs) [96]. Another study showed the generation of increased oxidative stress due to the viral induction of COX-2 [97]. One more report revealed that arachidonic acid metabolism by COX-2 is likely to produce ROS in human intestinal epithelial cells [98]. On the other hand, evidence has suggested the generation of oxidative stress through the inhibition of COX-2. For example, the COX-2 level was diminished due to the inhibition of aldose reductase (AR), which subsequently led to a decreased proliferation of human colon cancer cells through the inhibition of NF-κB and protein kinase C (PKC) [99]. Another very interesting report demonstrated that the level of COX-2 may be determined by the amount of ROS in the tumor milieu. According to this report, increased proliferation of HT-29 was evident with an increased level of COX-2 and vice versa [100]. Another study showed that pterostilbene (PS) treatment was associated with a reduction in oxidative markers NOS, COX-2, AR, and NF-κB and an increase in antioxidant glutathione reductase in an azoxymethane (AOM)-induced colon cancer model. This was also associated with a decrease in AOM-induced formation of aberrant crypt foci (ACF), lymphoid nodules (LNs), and tumors [101]. Further studies are recommended to understand the underlying molecular mechanisms of COX-2 signaling in CRC.

### 3.2. Transcription Factors

Apart from the signaling pathways, several transcription factors have also been reported to be ROS-sensitive and redox modification of these transcription factors can be instrumental in the initiation of CRC.

p53 protein, being the guardian of the genome, is central to orchestrate the cell cycle arrest, DNA repair, and apoptosis [102]. The cysteine residues in the DNA-binding domain of p53 protein are highly susceptible to oxidation and can be converted to sulfenic acid (PSOH), sulfinic acid (PSO_2_H), and finally sulfonic acid (PSO_3_H) by ROS in a stepwise reaction [103]. Mutagenesis of cysteines 176, 238, and 242 abrogates DNA binding, and substituting cysteines 124, 135, 141, and 277 obstructs the structural dynamics of DNA binding domain (DBD) [104,105]. Moreover, cysteines 182 and 277 were involved in redox regulation of p53 protein, and cysteines 176, 182, 238, and 242 were reported to be oxidized when treated with H_2_O_2_ [72]. Redox-mediated alteration in p53 protein may be associated with the development of colorectal carcinogenesis [106]. The transcriptional activity of p53 was downregulated by oxidation that promoted CRC by inhibiting apoptosis [107,108,109]. Another study reported that S-glutathionylation on Cys141 residue inactivates p53 protein that facilitates colon carcinogenesis [110].

The regulation of another redox-sensitive transcription factor, Nrf2, is disrupted in CRC that renders chemoresistance [111]. Nrf2, upon translocation to the nucleus, binds to antioxidant responsive element (ARE) and induces antioxidant response [58,112]. Nrf2-mediated response to ROS suppresses hypoxia-inducible factor-1 alpha subunit (HIF-1α)-VEGF signaling, thereby results in decreased growth and angiogenesis in CRC [113]. This is worth mentioning that in different cancers, an elevated Nrf2 activity is reported that bestows malignant cells highly proliferative, resulting in chemoresistance and negation of the usual role of Nrf2 [114,115]. Nevertheless, further studies are required to identify whether similar behavior of Nrf2 prevails in CRC.

NF-κB is another vital transcription factor that is involved in numerous physiological functions such as metabolism, inflammation, cell cycle, and apoptosis. This transcription factor is also implicated in the CRC [116]. ROS-induced deregulation of NF-κB is reported to be associated with carcinogenesis and chemoresistance [117]. The thiols of NF-κB are susceptible to redox modification. ROS induce the formation of an intrachain disulfide bond between Cys54 and Cys347 of nuclear factor-kappa B essential modulator (NEMO), the regulatory subunit of the inhibitor of kappaB kinase (IKK) complex. This leads to the phosphorylation of NF-κB inhibitory protein (IκB) and the release of NF-κB that later undergoes nuclear translocation [118]. Paradoxically, activation of NF-κB can be suppressed by ROS through the S-glutathionylation on the Cys189 of IKKβ [119].

Hypoxia-inducible factor-1 alpha subunit (HIF-1α), a transcription factor usually induced in hypoxic conditions, has been demonstrated to contribute to angiogenesis and metastasis in several cancers including CRC [120,121]. Basically, metastasis is promoted by several factors that get altered in hypoxia and these factors include Beclin 1, a vascular endothelial growth factor receptor along with eukaryotic initiation factor 5A isoform 2 (EIF5A2), phosducin-like 3, etc. [122,123,124]. Hypoxia promotes aggressiveness in CRC, which ultimately results in drug resistance, metastasis, and poor prognosis [125]. HIF-1α in CRC is activated by redox modification as evidenced by Schmitz et al. According to their report, redox-mediated translocation of HIF-1α into the nucleus enabled the expression of its target genes that facilitated the development of CRC [126]. Another study revealed that HIF-1α is a potential target for S-nitrosation that led to an amplified transcriptional activity by recruiting p300 co-activator protein to the HIF-1α C-terminal domain [127]. Additionally, S-nitrosation lowered HIF-1α ubiquitination, which indirectly favored angiogenesis in CRC [128].

## 4. The Role of Tumor Microenvironment (TME) in ROS Production and Their Pathophysiological Impact

Previous research confirmed that the TME plays a central role in the development of an adenomatous polyp that eventually leads to an invasive colon carcinoma [129]. In fact, TME serves as a hallmark of cancer and a better understanding of the generation of ROS by the component cells of the TME is critical to enhance therapeutic options and clinical outcomes.

Hypoxia, a salient feature of TME, is characterized by an imbalance between increased oxygen consumption and inadequate oxygen supply [130]. The overexpression of HIF-1α protein is reported in multifarious solid malignancies including colon cancer [131]. The tumor cells exposed to hypoxia harbor increased levels of ROS [132]. The hypoxic tumor cells exhibit adaptation for their survival by upregulating antioxidant capacity and result in more invasive and chemoresistant phenotypes [133,134]. Gao et al. reported that vitamin C and N-acetyl cysteine-mediated antitumor effects are HIF-1-dependent in the murine models of Myc-mediated tumorigenesis [135]. Essentially, ROS can stabilize HIF-1α under basal oxygen condition as illustrated by Haddad et al. where they reported that cytokine-mediated HIF-1α stabilization and activation entails a ROS sensitive mechanism [136]. Schmitz et al. also reported a similar finding where they showed that redox modification of HIF-1α promoted its target gene expression that ultimately resulted in the development of CRC [126].

Apart from hypoxia, other cells of TME such as cancer-associated fibroblasts (CAFs) also furnish ROS that can impact the pathology of cancer [137]. CAFs harbor heightened levels of H_2_O_2_ that emerge due to a disruption in transforming growth factor beta (TGF-β) signaling [138,139]. This aberrant TGF-β signaling leads to an elevated production of intracellular ROS by impairing mitochondrial function and inhibiting GPx1 and overproduction of extracellular ROS by inducing NOX [140]. CAF-induced extracellular ROS generation is also augmented by Caveolin-1 that serves as a negative regulator of NOX-derived ROS [141]. Upon exposure to H_2_O_2_ or CAF-conditioned medium, normal fibroblasts get transformed into an oxidative, CAF-like state that possesses increased CAF biomarkers, namely, fibroblast activation protein (FAP) and α-smooth muscle actin (αSMA) [142].

Myeloid cells, being a major component of inflammatory reactions, are associated with the regulation of tumor-associated immune suppression, tumor growth, and metastasis. [143]. In cancer, myeloid cells can be either mature cells such as macrophages and granulocytes or pathologically activated immature cells like myeloid-derived suppressor cells (MDSC) [144]. While their exact molecular mechanisms in cancer are yet to be clarified, a major mechanism is their ability to produce and release ROS. Reports showed that myeloid cell infiltration results in increased mutations due to ROS-induced DNA damage and myeloid cell-derived ROS might promote mutations, leading to an elevated risk of colon cancer in patients with IBD [145,146]. Canli et al. demonstrated that myeloid cell-derived H_2_O_2_ promotes invasive growth to intestinal epithelial cells through DNA mutations [147]. MDSC-derived peroxynitrite was found to disrupt the recruitment of cytotoxic T cells to tumors. Moreover, MDSCs from NOX2-deficient mice showed deregulated ROS generation, diminished production of interferon gamma (IFNγ), and impaired suppression of T cell proliferation [148,149].

In the TME, tumor-associated macrophages (TAMs) are another class of mediators of inflammation and tumorigenesis. ROS are closely linked to macrophage activation and signaling. Kraaij et al. reported that ROS generated from macrophages induced Tregs, indicating their immunosuppressive effects [150]. TAMs-derived ROS promotes apoptosis in lymphocytes through immune alterations [151]. They can generate ROS by producing redoxosomes that are NOX-enriched exosomes [152]. Another study showed that TAMs can promote ROS generation within cancer cells through tumor necrosis factor alpha (TNFα) secretion [153].

## 5. The Role of ROS in Tumor Metastasis through Epithelial–Mesenchymal Transition (EMT)

Metastasis refers to a complex, multi-step process characterized by the spreading of the primary tumor cells through EMT to distant organs [154,155]. Classically, EMT is marked by the acquisition of an invasive tumor phenotype that gets detached from the basement membrane with an ability to migrate and extravasate distant organs [156,157]. Certain transcription factors namely, SNAIL, Twist, and Zinc finger-E-box-binding (ZEB) serve as the master regulators of promoting the mesenchymal phenotype [158].

The existing evidence displays a strong link between the EMT of epithelial cancer cells and ROS. For example, SNAIL overexpression is associated with an augmented expression of Wnt target genes in CRC cell lines [159]. Jiao L and colleagues showed that heightened levels of ROS upregulate EMT via activation of SNAIL [160]. ROS leads to the stabilization of HIF-1α by inhibiting the activity of prolyl hydroxylase (PHD), which results in the transcription of SNAIL and Twist. An enhanced level of ROS can also activate NF-κB signaling that is strongly linked to the EMT process [161]. As mentioned before, NF-κB gets activated when the IKK complex phosphorylates IκB, leading to the ubiquitination and subsequent degradation of IκB by the 20S proteasome. Activated NF-κB, upon translocation to nucleus, induces transcription of different target genes [162]. Indeed, by inducing IKK-mediated degradation of IκB, ROS can facilitate the nuclear translocation of NF-κB that consequently promotes the transcriptional activation of SNAIL, Slug, Twist, and ZEB1/2 [161]. Studies showed that ROS-mediated tumor migration is facilitated by hypoxia-induced cathepsin and matrix metalloproteinase (MMPs) expression [163,164]. Another study reported that ROS promotes cell migration and invasion through uPA (urokinase-type plasminogen activator) and MMP-9 that are regulated by TGF-β1 [165]. The ROS exert a pivotal role in the regulation of EMT via the TGF-β1-TGF-β-activated kinase 1 (TAK1) pathway [166]. Matsuno et al. demonstrated that TGF-β1-induced EMT is mediated through ROS-Nrf2 pathway with Notch signaling [167].

Collectively, ROS display multifaceted roles in EMT and increasing evidence demonstrates that ROS are involved in numerous pathways that underscore their significance and indispensable roles in promoting EMT.

## 6. Development of Drug Resistance Due to Redox Adaptation in CRC

Drug resistance is a huge deadlock for which CRC has emerged as an arduous disease to treat. Tumor cells have a tendency of gaining an adaptive response against persistent ROS stress and such type of redox adaptation results in drug resistance over the course of therapy. Several mechanisms have been proposed behind redox adaptation that may include an elevated expression of antioxidants such as glutathione and SOD, upregulation of efflux transporters, enhanced DNA repair capability, inhibition of apoptotic machinery like caspases, and increased expression of cell survival molecules such as B-cell lymphoma 2 (Bcl-2) [168]. Another crucial element that imparts redox adaptation is ROS-induced DNA mutation. These mutations entail considerable genomic instability that serves as the secondary basis of redox adaptation in tumor cells [169]. Consequently, cancer cells can tolerate a heightened level of ROS stress and promote senescence evasion, angiogenesis, and metastasis. Taken together, aberrant drug metabolism in concert with the upregulation of cell survival mechanisms render chemoresistance to cancer cells. Therefore, understanding the tumor redox biology in terms of intrinsic and acquired therapy resistance would be fascinating to develop novel redox modulating anticancer agents.

Turkington et al. demonstrated that fibroblast growth factor receptor 4 (FGFR4) induces chemoresistance in CRC by regulating Flice inhibitory protein (FLIP) expression [170]. Indeed, the expression of FLIP is downregulated with the loss of S-nitrosylation that ultimately leads to the activation of apoptotic machinery [171]. Overexpression of SOD varieties, namely, SOD1 and SOD2, was shown to render detoxification to oxaliplatin-resistant colon cancer cells [172]. These oxaliplatin-resistant colon cancer cells harbor elevated expression of Nrf2 that is also associated with chemoresistance [173]. An increased level of intracellular ROS was evident in colon cancer cells that are 5-fluorouracil (5-FU) resistant [174]. Dual oxidase (DUOX), a form of NOX, is overexpressed in 5-FU chemoresistant colon cancer cell lines and their enhanced expression enables the tumor with invasion ability [175]. Indeed, NOX expression that is linked to the regulation of cell growth and proliferation was shown to be upregulated in colon cancer [176]. All these studies indicate that redox adaptation has a prominent role in developing resistance to currently available chemotherapeutics and, hence, a more in-depth investigation is required to explore the exact role of ROS in CRC drug resistance [177].

## 7. Counteractive Defense and Prevention of ROS from Occurring

There are numerous cellular antioxidants such as GPx, GR, SOD, catalase, etc. that are the major sources of defense against oxidative stress. DNA repair proteins are also involved in this defense mechanism. They encompass endo- and exonucleases, glycosylases, DNA ligases, DNA polymerases, and so forth. For example, DNA glycosylases participate in the repair and removal of the oxidized base containing DNA mainly through the base excision repair (BER) [38]. Other types of oxidative lesions are repaired by nucleotide excision repair (NER) and mismatch repair (MMR) [45]. The initiation of CRC is closely associated with a disruption in these repair proteins. Genetic alterations like single-nucleotide polymorphisms (SNPs) were observed in SOD2, myeloperoxidase, and eosinophil peroxidase genes [178]. Another report showed a correlation between selenoprotein gene modification with CRC. According to this report, SNPs in selenoprotein P plasma 1 (SEPP1), glutathione peroxidase 4 (GPx4), and selenoprotein S (SELS) genes had demonstrated a greater risk of CRC development [179]. A similar pattern of risk was confirmed in another study that had displayed SNPs in SEP15 and SELS genes with a concomitant risk of CRC [180]. Moreover, a disparity in the MAPK signaling pathway was also found to be associated with elevated CRC risk [181]. Other studies also lent support on mutation in selenoprotein genes with CRC development [182]. From the perspective of micronutrients, vitamins C and E were found in a diminished amount in CRC patients [183]. In GPx-3-deficient mice, inflammatory colon carcinoma was evident with increased inflammation, proliferation, and DNA damage [184].

The goal of counteracting oxidative-stress-induced damage should not compromise the well-integrated antioxidant defense network. Hence, to prevent oxidative stress, the approach should be the prevention of free radical formation. The micronutrients are beneficial in the redox homeostasis as ROS scavengers. They include beta-carotene, vitamin C, vitamin E, and so on [185]. Yun et al. reported selective tumor cell killing by vitamin C in colorectal tumor cells having KRAS or BRAF mutations [186]. Consumption of vegetables and fruits is associated with the eradication of free radicals. Apart from these, foods enriched with omega-3 fatty acids (EPA—Eicosapentaenoic acid and DHA—Docosahexaenoic acid) are also advantageous as they can stabilize mitochondrial membranes [187,188,189]. A growing body of evidence lends support to the hypothesis that caloric restriction may decrease oxidative stress [190,191]. This caloric restriction results in a rapid and more efficient electron transport that may generate diminished levels of mitochondrial ROS due to a reduction in electron leakage from the respiratory chain [192,193]. Certain agents may function as calorie restriction mimetics that may render the benefits of caloric restriction instead of restricting diet strictly [194]. These include agents that promote the action of insulin (e.g., metformin), polyphenolic compounds obtained from the plants (e.g., butein, quercetin, and piceatannol), or agents that inhibit glycolysis (e.g., 2-deoxyglucose) [195]. Chelation of transition metals prevents them from generating free radicals in the cells and, thereby, confer antioxidant property. For example, copper- and iron-catalyzed Fenton reactions produce free radicals that may be inhibited by albumin, transferrin, and ceruloplasmin, which are metal-chelating antioxidants. Transferrin and ferritin also sequester iron [196].

## 8. Targeting Redox Alterations in CRC

**Figure 3:**
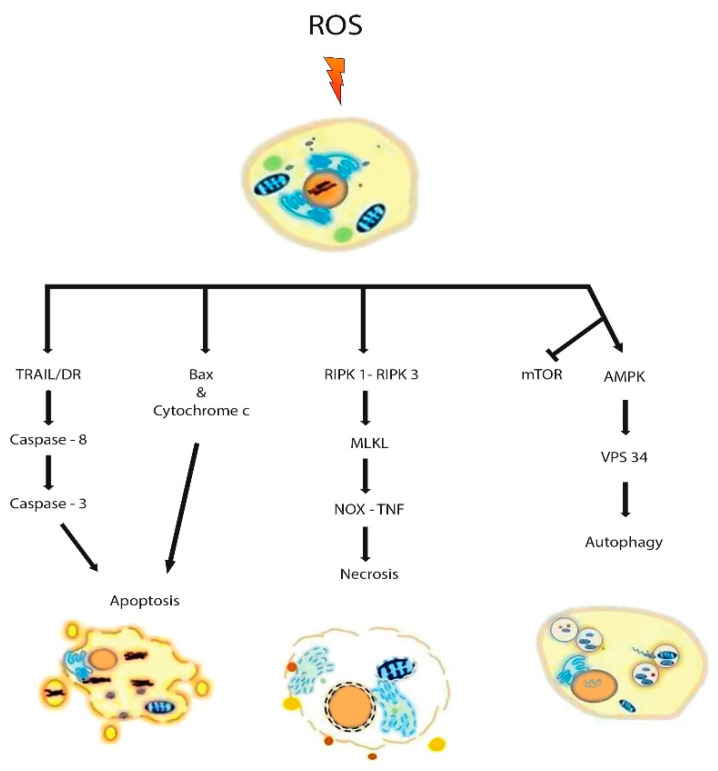
Mechanisms of ROS-mediated cancer cell death. Elevated levels of ROS can orchestrate cancer cell death by apoptosis, necrosis, and autophagy. ROS-mediated activation of Bax, death receptors (DR), and caspases elicits apoptotic cell death. Increased oxidative stress can also lead to necrosis by forming receptor interacting protein kinase, RIPK-1/RIPK-3 complex. Moreover, NOX can interact with TNF to facilitate necrosis. Redox stress-induced inhibition of mammalian target of rapamycin (mTOR) activity and activation of AMP-activated protein kinase (AMPK) stimulate vacuolar protein sorting 34 (VPS 34) complex that results in the commencement of autophagy.

Extensive research has been undertaken to exploit the heightened oxidative stress in tumor cells through ROS-mediated mechanisms. ROS-inducing agents may induce apoptosis, necroptosis, and autophagy (Figure 3). Two pathways are typically responsible for the induction of apoptosis, namely, intrinsic and extrinsic pathways, and both of these are regulated by caspases. ROS-mediated activation of BH3 interacting-domain death agonist (BID), Bax, caspases, and death receptors (DR) orchestrate apoptotic cell death. In the intrinsic pathway, caspase-9 promotes the activation of apoptotic inducer caspase-3 and in the extrinsic pathway, caspase-8 mediates the activation of caspase-3. The intrinsic pathway is governed by endoplasmic reticulum (ER) and mitochondria and the extrinsic pathway is directed by DRs [197]. Necroptosis generally occurs when the scarcity of adenosine triphosphate (ATP) suppresses apoptosis [198]. The process of necroptosis is distinct from apoptosis since it does not depend on the activation of caspases, rather depends on the kinase receptor-interacting protein (RIP) family, such as RIP-1 and -3 [199]. Intriguingly DRs are also involved in the regulation of necroptosis [200]. Autophagy encompasses a process of cellular homeostasis through the digestion of superfluous intracellular contents via lysosomal degradation. In this process, H_2_O_2_ oxidizes autophagy related 4A cysteine peptidase (ATG4) that results in the lipidation of microtubule-associated protein 1 light chain 3, LC3/ATG8 [201]. Simultaneously, redox stress inhibits mTOR that leads to the stimulation of vacuolar protein sorting 34 (VPS34) by activating AMP-activated protein kinase (AMPK) [202].

**Figure 4:**
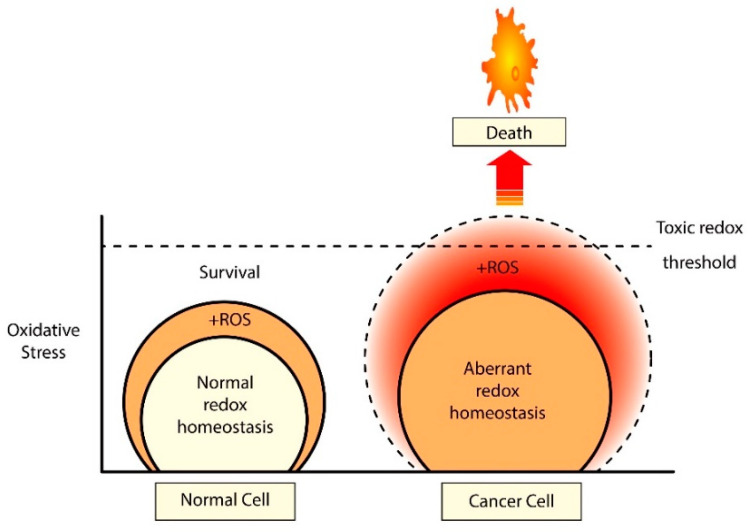
Exploitation of elevated oxidative stress for therapeutic selectivity. Tumor cells including colorectal cancer (CRC) cells harbor heightened oxidative stress that might serve as the Achilles’ heel for their preferential elimination. Normal cells maintain a decent balance of ROS generation and elimination and can use their regular antioxidants to counteract excessive ROS. However, due to their increased reliance on the antioxidants, tumor cells become vulnerable to further ROS insults. Hence, the application of ROS-generating agents may overwhelm the intracellular antioxidants of tumor cells by forcing them beyond the toxic redox threshold that can drive tumor cell apoptosis. This unique feature of tumor cells serves as the basis of therapeutic selectivity for their preferential elimination.

Therapeutic selectivity is instrumental to devise effective anticancer therapeutics. Owing to their inherent elevated oxidative stress, cancer cells are heavily reliant on antioxidants for their survival that makes them increasingly vulnerable to exogenous ROS modulating agents, as illustrated in Figure 4 [203,204]. Hence, it is conceivable to induce selective tumor cell killing by manipulating their endogenous redox states, and a huge number of studies deployed both natural and synthetic ROS-modulating agents to combat CRC (Table 1).

5-Fu has been used in the treatment of CRC for several decades. Beside its main mechanism of action of inhibiting the DNA synthesis, 5-Fu generated plenty of O^2•−^ that promoted CRC cell apoptosis by positively regulating the p53 proteins as evidenced by an in vitro study [205]. WZ35, a synthetic curcumin derivative, promoted the generation of ROS and induced caspase-dependent apoptosis. WZ35 also displayed robust antitumor effects in CT26 xenograft model through ROS-induced ER stress-dependent apoptosis [241]. Andrographolide, a plant-derived compound, was reported to exert efficacy in a CRC cell culture model by inhibiting the progression of cell cycle and initiating ROS/ER-dependent apoptosis [207]. Basak et al. evidenced that another natural compound piperlongumine (PL) was able to augment the drug sensitivity of certain p53 mutant colon cancer cells through ROS-mediated functional restoration of p53 protein. Moreover, the compound also demonstrated significant tumor growth inhibition in HT-29 xenograft in nude mice [236]. Zeriouh et al. showed an increased ER stress-mediated apoptosis in CRC cell lines after treating them with the phenolic extract of the oleaster leaves (PEOL). Concurrently, they reported tumor growth suppression in HCT116 xenograft in nude mice by PEOL [234].

ERK-dependent activation-induced cell death was demonstrated by certain ROS-inducing agents. For example, 2α,3α,24-thrihydroxyurs-12-en-28-oicacid (TEOA), obtained from *Actinidia eriantha* roots, upregulated autophagy in SW620 cells through ROS/ER-dependent pathway. Additionally, the compound also reduced tumor burden in SW620 xenograft model [239]. HMF treatment of HCT116 cells exhibited DNA damage with a compromise in mitochondrial membrane potential that was followed by cytochrome c release and activation of apoptotic machinery. The study also demonstrated an upregulation of mitochondrial and cytosolic ROS generation with a concomitant decrease in antioxidant enzyme expression. The effects of HMF treatment were reversed by pre-treatment with the ROS scavenger N-acetyl-l-cysteine (NAC), indicating the significance of ROS in the anticancer effects of HMF [229]. The impact of ROS could be accentuated from a study where Lim et al. revealed that hispidin, a phenolic compound, induced apoptosis by virtue of promoting ROS in colon cancer cells. This drug diminished cell viability both in mouse and human colon cancer cells. Furthermore, hispidin-induced apoptosis was blocked in the event of a pre-treatment with the ROS scavenger, NAC [228]. Han et al. reported that HCT116 cells underwent apoptosis when treated with a benzophenanthridine alkaloid, sanguinarine and this was implemented by ROS-mediated Egr-1 activation and mitochondrial dysfunction [238]. Another study demonstrated that bufalin promoted autophagy in HT-29 and Caco-2 cells through ROS-activated autophagy via the c-Jun NH(2)-terminal kinase (JNK) [212]. 2- Chloro-1,4-naphthoquinone (CHNQ), a quercetin derivative, also displayed ROS-mediated autophagy in HT-29 and HCT116 cells as evidenced by the buildup of acidic vesicles, a marked increase in the lipidation of LC3, and decreased activation of protein kinase B (AKT) [217]. A recent study showed that CJK-7, a novel flavonoid, induced ROS-mediated autophagy in HCT116 cells via JNK as well [219].

Pathi et al. described a novel ROS-generating agent ethyl 2-((2,3-bis(nitrooxy)propyl)disulfanyl)benzoate (GT-094) that comprises a nonsteroidal anti-inflammatory drug (NSAID) and NO moieties. GT-094 treatment in CRC resulted in elevated apoptosis accompanied by induction of reactive oxygen species (ROS), and these effects were reversed after cotreatment with the antioxidant glutathione. The drug also repressed Sp1, Sp3, and Sp4 regulated gene products by downregulating microRNA-27a (miR-27a) [227]. Resveratrol was shown to induce caspase-mediated apoptosis via autophagy that correlated with the induction of ROS in HT-29 and COLO201 cells [237]. A recent report also lends support to the ROS-mediated apoptosis where citral, an acyclic monoterpene, was found to augment intracellular ROS in HCT116 and HT-29 cells followed by apoptosis [218]. A similar result was observed when HCT116 cells were treated with Levistolide A that induced ROS-mediated apoptosis by promoting ER stress [231]. Agarwal et al. reported similar ROS-mediated apoptosis and cell cycle inhibitory effects in HT-29 cells by curcumin [222]. Dihydrotanshinone I (DHTS) displayed both ROS-mediated apoptosis and autophagy in HCT116 cells as well as in HCT116 xenograft setting that was developed in nonobese diabetic/severe combined immunodeficient (NOD/SCID) mice [223]. Meanwhile, ziyuglycoside II, a major bioactive compound, revealed an altered production of ROS followed by both caspase-dependent and independent apoptosis in HCT116 cells [242]. Avenanthramide A induced ROS-mediated apoptosis by inhibiting the expression of DDX3, an RNA helicase in HCT116 and DLD-1 cells. This molecule also vigorously decreased tumor growth in DLD-1 xenograft in nude mice [208]. Very recently, D’Onofrio et al. reported that δ-Valerobetaine (δVB), a milk metabolite, induced ROS-mediated apoptosis in LoVo cells [233].

Several studies demonstrated a central role of ROS in the DR/TNF-related apoptosis-inducing ligand (TRAIL)-induced apoptosis [213,243]. Casticin, a compound obtained from Vitex rotundifolia, leads to TRAIL-induced apoptosis where ROS inhibits the expression of anti-apoptotic proteins such as Bcl-2, Bcl-xL, X-linked inhibitor of apoptosis protein (XIAP), and survivin [215]. The compound also resulted in ROS-induced apoptotic cell death in COLO205 [216]. A similar kind of effect was noted with the treatment of a resveratrol analog, bakuchiol in CRC as the event was associated with TRAIL-induced apoptosis as well as an increase in the DR4 expression [209]. Previously, Su et al. reported that treatment of HCT116 cells with 15dPGJ(2) sensitized them to TRAIL-induced cytotoxicity through the upregulation of DR5. They concluded that the upregulation of DR5 by 15dPGJ(2) resulted via ROS induction [206]. Aberrant histone deacetylase (HDAC) activity is associated with tumor development and some histone deacetylase inhibitors (HDACIs) are reported to synergize with TRAIL in different human cancers [244]. Recently, Huang et al. demonstrated that droxinostat, a HDACI, also enhanced ROS production followed by apoptosis in HT-29 cells. However, the role of TRAIL in such type of apoptosis is yet to be investigated [225].

Specificity protein (Sp) transcription factors (Sp1, Sp3, and Sp4) that are vital in tumor development and metastasis are overexpressed in colon and other cancer cell lines [245,246,247]. Some common Sp-regulated genes include EGFR, Bcl-2, survivin, VEGF, and p65 subunit of NF-κB [248,249,250]. One study identified that a naturally occurring triterpenoid, betulinic acid (BA) elicited repression of Sp1, Sp3, and Sp4 along with microRNA-27a by generating ROS in RKO cells and RKO xenografted tumors in athymic nude mice [210].

Disruptions of several pathways (e.g., MAPK, PI3K/AKT, JAK/STAT, Wnt signaling pathways) are implicated in the development of CRC. A diverse array of mutations in the components of these signaling pathways present a major obstacle to shifting the balance to ROS-mediated apoptosis [251]. Hence, the modulation of intracellular ROS signaling to suppress the tumor-promoting events within these pathways may hold the immense prospect for developing better anti-cancer treatment strategies. Indeed, the chemotherapeutics that work by inducing elevated ROS may be dependent on the stage and endogenous ROS level in the tumor and the abundance of ROS-promoted survival pathways. Two novel treatment strategies may be of interest beyond the activation of relevant pathways in CRC, and more comprehensive studies are warranted to confirm their efficacy. The first one is photodynamic therapy (PDT) that utilizes the light and photosensitizer, which converts surrounding oxygen into ROS under the irradiation of a specific wavelength of light [252]. The increased levels of ROS elicit tumor cell death by damaging the DNA and promoting apoptosis [253]. Chen et al. demonstrated a novel approach to vanquishing hypoxia in solid tumors by designing a cell-specific nanoparticle that was able to generate O_2_ and be used for PDT. In this study, they showed that with the entry of nanoparticles into the tumor cells, the intracellular H_2_O_2_ gets converted to O_2_, which ultimately results in the elimination of tumor cells [254]. This is important to mention that ionizing radiation therapy can also generate ROS that results in DNA damage and cell death [255]. As evidenced by Yoshida et al., exposure to ionizing radiation resulted in a rapid and vigorous induction of OH^•^ that oxidized the electron transport chain (ETC) complex, leading to mitochondrial dysfunction and eradication of CRC cells [256]. The second strategy is the exploitation of ROS in immunotherapy that essentially employs T lymphocytes or natural killer (NK) cells to eradicate tumor cells. Habtetsion et al. reported redox-dependent CRC cell death by altering tumor metabolism through CD4^+^ T cell-mediated adoptive immunotherapy. They found a compromised antioxidant capacity of the TME due to the accumulation of excessive ROS that led to their DNA damage [257]. Ligtenberg et al. demonstrated another novel way of promoting tumor cell killing by making antitumor T cells more robust against ROS. Their study paved a method of sustained antitumor activity under hypoxia and oxidative stress through the adoptive transfer of T cells [258].

## 9. Concluding Statements and Future Perspectives

Like other cancers, CRC cells harbor elevated levels of ROS that promote cell proliferation and, in advanced stages, are coupled to redox adaptation that facilitates cell survival and drug resistance. However, further investigation is required to understand the comprehensive redox biology of CRC in terms of its initiation, progression, and/or response to therapy. The exploitation of the heightened oxidative stress of tumor cells through redox modulation holds an immense potential to preferentially eliminate these tumor cells while sparing their normal counterparts. A good number of ROS enhancers displayed promising therapeutic selectivity in CRC cells in preclinical models by inducing oxidative stress over the toxicity threshold. Indeed, an inhibitor of ROS eliminator together with a ROS enhancer might be an attractive combination approach to promote ROS accumulation in tumor cells, thereby amplify selective toxicity. At the same time, the combination of an inhibitor of redox adaptation with standard chemotherapeutics could be another avenue of further investigation to improve therapeutic outcomes in CRC. Hence, based on our overall discussion, it is explicitly evident that the redox modulatory approach could have major implications in CRC treatment.

## Figures and Tables

**Table 1:** ROS-inducing agents used in CRC-derived cell lines.

Compounds	Cell Lines	Major Outcomes	Mechanisms	References
5-FU	HT-29	↑ Caspase-7, Src	ROS-dependent apoptosis	[205]
15dPGJ (2)	HCT116, SW480	↑ CHOP, GRP78, XBP1	ROS/TRAIL-dependent apoptosis	[206]
Andrographolide	T84, COLO205	↑ Nrf2, GPx, PrX-6, LPO, TRX; ↓ ΔΨm	ROS/ER/caspase dependent apoptosis	[207]
Avenanthramide A	HCT116, DLD-1	↑ Caspase-3, Cyto c	ROS-mediated apoptosis	[208]
Bakuchiol	HCT116, HT-29	↑ Caspase-3, -8, -9, PARP; ↓ Bcl-2, survivin, cFLIP, XIAP	ROS/DR-dependent apoptosis	[209]
Betulinic acid	RKO, SW480	↓ Sp, microRNA-27a, ZBTB10 gene	ROS-apoptosis	[210]
Benzimidazole acridine derivative	HCT116, SW480	↑ Caspase-3, -7, -8, -9, Bid, PARP; ↓ Bcl-2	ROS/DR-dependent apoptosis	[211]
Bufalin	HT-29, Caco-2	↑ LC3-II, ATG5, Beclin-1	ROS-autophagy	[212]
Capsazepine	HCT116, HT-29	↑ Caspase-8, -9, Bax; ↓ cFLIP, survivin	ROS/DR-dependent apoptosis	[213]
Cardamonin	HCT116	↑ Cleaved PARP, caspase-8, -9, -3, Bax; ↓ cIAP-1, cFLIP, XIAP, Bcl-2	ROS /DR/TRAIL-dependent apoptosis	[214]
Casticin	HCT116, SW480, HT-29, COLO205	↓ Bcl-2, Bcl-xL, cFLIP, XIAP; ↑ CDKN1B gene, TRAP1 gene, G2/M phase arrest; ↓ ΔΨm, [Ca^2+^]i, MMP-2, RKAR2B gene, CaMK4 gene	ROS/DR-dependent apoptosis ROS/caspase-dependent apoptosis	[215] [216]
CHNQ	HCT116, HT-29	↑ LC3-II, puncta formation, acidic vesicle; ↓ AKT/PKB	ROS-autophagy	[217]
Citral	HCT116, HT-29	↑ phospho-p53, Bax, Cleaved caspase-3; ↓ Bcl-2, Bcl-xL	ROS-apoptosis	[218]
CJK-7	HCT116	↑ p53, Puma, ATG5, Beclin-1, LC3-I/II; ↓ Bcl-2	ROS-apoptosis and autophagy	[219]
CLA	SW480	↑ Phosphorylated eIF2α, Xbp1 mRNA, CHOP	ROS/ER/caspase dependent apoptosis	[220]
Compound K	HCT116	↑ Caspase-3, -9, LC3-II, flux ATG6, ATG7; ↓ Bcl-2	ROS-apoptosis and autophagy	[221]
Curcumin	HT-29	↑ S & G2/M arrest, DNA fragmentation; ↓ ΔΨm	ROS-apoptosis	[222]
DHTS	HCT116	↑ Bax, Bcl-xl, caspase-3, Cyto c, AIF, LC3-II	ROS/caspase-apoptosis and autophagy	[223]
DMF	HCT116, CT26, HT-29	↓ GSH	ROS-mediated necroptosis	[224]
Droxinostat	HT-29	↑ Acetylated H3, H4, caspase-3, Bax, Puma; ↓ HDAC3, 6, Bcl-2, Bcl-xl	ROS-apoptosis	[225]
Flavokawain B	HCT116	↑ Cyto c, GADD153; ↓ Bcl-2 family members	ROS-apoptosis	[226]
GT-094	RKO, SW480	↓ VEGF, MMP, c-Met, EGFR, Sp microRNA-27a	ROS-apoptosis	[227]
Hispidin	HCT116, CMT-93	↑ p53, Bax, caspase-3, -8; ↓ Bcl-2	ROS/DR/caspase-dependent apoptosis	[228]
HMF	HCT116	↑ [Ca^2+^]i, Cyto c, BID, Bax; ↓ Bcl-2	ROS/ER/caspase-dependent apoptosis	[229]
Ilimaquinone	HCT116	↑ Caspase-8, -3; ↓ Bcl-2, Bcl-xL	ROS/DR-dependent apoptosis	[230]
Levistolide A	HCT116	↑ Caspase-3, cleaved-PARP	ROS-apoptosis	[231]
MAM	HCT116, HT-29	↑ [Ca^2+^]i, RIP1/RIP3	ROS-dependent necroptosis	[232]
Milk δ-Valerobetaine (δVB)	LoVo	↑ Caspase-9, -3, Bax, Sirtuin6	ROS-mediated apoptosis	[233]
PEOL	HCT116, HCT-8	↑ [Ca^2+^]i, Cyto c; ↓ ΔΨm	ROS/ER/caspase-dependent apoptosis	[234]
Physalin B	HCT116	↑ Cleaved-PARP, p62; ↓ Caspase-3, LC3-II	ROS-autophagy	[235]
Piperlongumine	HT-29, SW620	↑ Cleaved caspase-3, PARP, Bax	ROS-apoptosis	[236]
Resveratrol	HT-29, COLO201	↑ Caspase-8, -3, LC3-II	ROS-apoptosis and autophagy	[237]
Sanguinarine	HCT116	↑ Caspase-3, -9; ↓ Bcl-2, XIAP, cIAP-1	ROS-apoptosis	[238]
TEOA	SW620	↑ p62, Cleaved-PARP, LC3-II	ROS/ER/caspase-dependent apoptosis	[239]
Vitamin C	RKO, SW480	↓ EGFR, VEGF, c-Met, VEGFR1, Sp	ROS-dependent apoptosis and necrosis	[240]
WZ35	CT26	↑ Cleaved-PARP; ↓ CyclinB1, Cdc2, MDM-2	ROS/ER/caspase 3-mediated apoptosis	[241]
Ziyuglycoside II	HCT116	↑ p53, cleaved-PARP, caspase-3, -7, -8, -caspase-9; ↓ Bcl-2	ROS-apoptosis	[242]

## Abbreviations

8-OHdG	8-hydroxy-2′-deoxyguanosine
8-oxodG	8-oxo-7,8-dihydro-2′-deoxyguanosine
ACF	Aberrant crypt foci
AMPK	AMP-activated protein kinase
AOM	Azoxymethane
APC	Adenomatous polyposis coli
AR	Aldose reductase
ARE	Antioxidant responsive element
αSMA	α-smooth muscle actin
ATG4	Autophagy related 4A cysteine peptidase
ATP	Adenosine triphosphate
Bcl-2	B-cell lymphoma 2
BER	Base excision repair
CAFs	Cancer-associated fibroblasts
CD	Crohn’s disease
COX-2	Cyclooxygenase – 2
CRC	Colorectal cancer
CSC	Cancer stem cells
dG	Deoxyguanosine
dGTP	Deoxyguanosine triphosphate
DR	Death receptor
DUOX	Dual oxidase
EGFR	Epidermal growth factor receptor
EMT	Epithelial–mesenchymal transition
ER	Endoplasmic reticulum
ERK	Extracellular signal-regulated kinase
ETC	Electron transport chain
FAP	Fibroblast activation protein
FGFR4	Fibroblast growth factor receptor 4
FLIP	Flice inhibitory protein
GIT	Gastrointestinal tract
GPx	Glutathione peroxidase
HDAC	Histone deacetylase
HDACIs	Histone deacetylase inhibitors
HIF-1α	Hypoxia inducible factor 1 alpha subunit
HNE	4-hydroxy-2-nonenal
IBD	Inflammatory bowel diseases
JAK	Janus kinases
JNK	c-Jun NH(2)-terminal kinase
Keap1	Kelch-like ECH-associated protein 1
LCN-2	Lipocalin-2
LN	Lymphoid nodules
MAPK	Mitogen-activated protein kinase
MDA	Malondialdehyde
MDSC	Myeloid-derived suppressor cells
MMP	Matrix metalloproteinase
MMR	Mismatch repair
MTHFD2	Methylenetetrahydrofolate dehydrogenase
MYH	MutY homologue
NAC	N-acetyl-l-cysteine
NADPH	Nicotinamide adenine dinucleotide phosphate
NEMO	Nuclear factor-kappa B essential modulator
NER	Nucleotide excision repair
NF-κB	Nuclear factor kappa-light-chain-enhancer of activated B cells
NOD/SCID	Non-obese diabetic/Severe combined immunodeficient
NO-NSAIDs	Nitric oxide-releasing non-steroidal anti-inflammatory drugs
NOS	Nitric oxide synthase
NOX	Nicotinamide adenine dinucleotide phosphate oxidase
NOXA1	NOX Activator 1
NOXO1	NOX Organizer 1
Nrf2	Nuclear factor erythroid 2–related factor 2
NRX	Nucleoredoxin
OGG1	8-oxoguanine DNA glycosylase 1
PDGF	Platelet-derived growth factor
PDT	Photodynamic therapy
PG	Prostaglandin
PHD	Prolyl hydroxylase
PI3K	Phosphatidylinositol 3-kinase
PKC	Protein kinase C
PMA	Phorbol 12-myristate 13-acetate
PPP	Pentose phosphate pathway
PS	Pterostilbene
PTEN	Phosphatase and tensin homolog
PTP	Protein tyrosine phosphatase
PUFAs	Polyunsaturated fatty acids
RIP	Receptor interacting protein
RNS	Reactive nitrogen species
ROS	Reactive oxygen species
RTK	Receptor tyrosine kinase
SNPs	Single-nucleotide polymorphisms
SOD	Superoxide dismutase
Sp	Specificity protein
STAT	Signal transducer and activator of transcription proteins
TAK1	TGF-β1-TGF-β-activated kinase 1
TAMs	Tumor-associated Macrophages
TGF-β	Transforming growth factor beta
TIGAR	TP53-induced glycolysis and apoptosis regulator
TME	Tumor microenvironment
TRAIL	TNF-related apoptosis-inducing ligand
Tregs	Regulatory T cells
UC	Ulcerative colitis
uPA	Urokinase-type plasminogen activator
VEGF	Vascular endothelial growth factor
VPS34	Vacuolar protein sorting 34
Wnt	Wingless-related integration site
XIAP	X-linked inhibitor of apoptosis protein
ZEB	Zinc finger-E-box-binding
